# Assessing COVID-19 Vaccination Uptake and Incident Infections Among Ethiopian Healthcare Workers, Addis Ababa—2022: Implications for Public Health Preparedness

**DOI:** 10.1093/cid/ciaf341

**Published:** 2025-07-29

**Authors:** Gadissa Bedada Hundie, Tigistu Adamu Ashengo, Stacie C Stender, Mebratu Abraha, Abrham Getachew, Sisay Sirgu, Mahteme Bekele Muleta, Matthew Westercamp, Firew Ayalew

**Affiliations:** Department of Microbiology, Immunology and Parasitology, St. Paul's Hospital Millennium Medical College, Addis Ababa, Ethiopia; Jhpiego, Baltimore, Maryland, USA; Jhpiego, Baltimore, Maryland, USA; Johns Hopkins Bloomberg School of Public Health, Baltimore, Maryland, USA; Department of Nursing, St. Paul's Hospital Millennium Medical College, Addis Ababa, Ethiopia; Department of Public Health, St. Paul's Hospital Millennium Medical College, Addis Ababa, Ethiopia; Department of Internal Medicine, St. Paul's Hospital Millennium Medical College, Addis Ababa, Ethiopia; Department of Surgery, St. Paul's Hospital Millennium Medical College, Addis Ababa, Ethiopia; Division of Healthcare Quality Promotion, Centers for Disease Control and Prevention, Atlanta, Georgia, USA; Jhpiego Ethiopia, Addis Ababa, Ethiopia

**Keywords:** COVID-19, vaccination, healthcare workers, Ethiopia, SARS-CoV-2 infections

## Abstract

**Background:**

The coronavirus disease 2019 (COVID-19) pandemic exposed global public health preparedness challenges. In Ethiopia, healthcare workers (HCWs) faced barriers to vaccination despite widespread availability. Here, we describe severe acute respiratory syndrome coronavirus 2 (SARS-CoV-2) infections and vaccine uptake among HCWs in Addis Ababa after the national vaccine rollout.

**Methods:**

A prospective cohort study enrolled 469 HCWs with baseline and 5 follow-up visits over 6 months (January 2022–July 2022). Nasopharyngeal swabs were tested using real-time reverse transcription polymerase chain reaction. Associations among participant characteristics, vaccination status, and SARS-CoV-2 infection were assessed using the Fisher exact test and odds ratios (ORs) with 95% confidence intervals (CIs).

**Results:**

At baseline, 57% (267 of 469) of participants were fully vaccinated, with physicians having higher uptake than other HCWs (82% vs 52%; *P* < .001). Vaccination was not associated with perceived risk (*P* = 1.00) or workplace exposure (*P* = .08). Over 6 months, 11% (53 of 469) experienced SARS-CoV-2 infection, all with mild symptoms and no hospitalizations. Infection was not associated with vaccination (*P* = .66) or occupational characteristics but was more likely in HCWs who felt safe from infection (OR = 2.05; 95% CI, 1.11–3.81; *P* = .02).

**Conclusions:**

Despite vaccine availability, uptake remained suboptimal among Ethiopian HCWs. Misalignment between perceived risk and vaccination decisions suggests the need for targeted interventions. Educational campaigns and community leader engagement may improve vaccine acceptance. The association between perceived safety and infection risk highlights the need for continued infection prevention measures.

## Teaser

We examined vaccination status and incident SARS-CoV-2 infections among Ethiopian healthcare workers. Despite widespread availability, coronavirus disease 2019 vaccine uptake was only 57%. Infections were mild, with no hospitalizations. Enhanced education on vaccine safety and infection risks is essential.

The coronavirus disease 2019 (COVID-19) pandemic was a profound test of national health systems, highlighting both strengths and weaknesses in global public health preparedness, surveillance, and coordinated response [1, 2]. As severe acute respiratory syndrome coronavirus 2 (SARS-CoV-2) infections and hospitalizations wane, there is an opportunity for the thoughtful assessment of relevant public health information to help guide healthcare and public health system improvement and mitigate the impact of the next public health emergency.

Ethiopia is a low-income country and one of the most populous in East Africa. As of May 2023, Ethiopia reported more than 500 000 COVID-19 cases and 7500 related deaths to the World Health Organization (WHO); however, reporting was likely limited by challenges in the availability and utilization of SARS-CoV-2 testing [3–5]. As of 13 March 2021, COVID-19 vaccines were available and actively promoted in Ethiopia. Despite this progress, vaccine uptake remained suboptimal, primarily due to limited public awareness and concerns about the vaccine's effectiveness and safety [6]. Additionally, vaccination rates among key groups, such as healthcare workers (HCWs) who are essential to the national response and face an increased risk of infection, have not been sufficiently described.

Here, we present findings on vaccine uptake and incident SARS-CoV-2 infections among frontline HCWs, identified as a higher-risk group due to their assignments in patient care areas with potential ongoing exposure to SARS-CoV-2, within a tertiary healthcare system in Addis Ababa, Ethiopia.

## METHODS

### Study Design

The Ethiopian Healthcare Worker Post-Vaccine Cohort was a prospective cohort study designed to describe SARS-CoV-2 infection, including asymptomatic and presymptomatic infections, in HCWs serving a tertiary referral care hospital in Addis Ababa, Ethiopia, after a national COVID-19 vaccine rollout. The study included questionnaires and nasopharyngeal swab testing conducted at baseline and scheduled follow-up visits over a period of approximately 6 months, from late January 2022 to mid-July 2022.

Recruitment occurred at St. Paul's Hospital Millennium Medical College (SPHMMC) and included the main hospital facility (700 beds); the Addis Ababa Burn, Emergency, and Trauma Hospital (200 beds); and a dedicated 350-bed COVID-19 center with high-dependency ventilator and intensive care. All HCWs at SPHMMC were offered the Oxford-AstraZeneca COVID-19 vaccine (ChAdOx1 nCoV-19), with the first dose offered in March 2021, the second dose in early July 2021, and booster doses offered on an ongoing basis as available. Vaccination was encouraged but not mandatory. During the study period, published SARS-CoV-2 sequencing data indicated that Omicron variants BA.1 and BA.4/5 were the predominant strains circulating in Ethiopia [7, 8].

### Study Participants

Adult (aged ≥18 years) HCWs with a potential ongoing risk of COVID-19 were invited to participate through staff meetings and information sessions. Participation was voluntary, and written informed consent was obtained before enrollment. HCWs with various roles and from various patient care areas were eligible to participate based on their work assignment. To minimize selection bias, recruitment efforts targeted a broad range of hospital departments to ensure diverse representation. No additional inclusion or exclusion criteria were applied.

### Baseline Visit

Participants completed a baseline questionnaire that covered occupation details, household characteristics, medical history (including select comorbid conditions, perceived health status, smoking history, and vaccination history), occupational exposure, previous COVID-19 symptoms, contact with SARS-CoV-2–infected individuals, and prior testing for SARS-CoV-2 infection. Participants were considered fully vaccinated if they received 2 or more doses of COVID-19 vaccine >14 days prior to recruitment.

### Follow-up

Following recruitment (ie, baseline visit), participants were engaged over a 6-month period for in-person, planned, bi-weekly follow-up visits that included collecting nasopharyngeal swabs and administering questionnaires to gather updated occupational information and COVID-19 vaccination status, details on new SARS-CoV-2 exposures and test results, and any symptoms compatible with COVID-19. All data were collected and managed using custom forms and databases through Standard Data (https://www.standardco.de).

Participants who missed a follow-up appointment for any reason resumed their follow-up upon return to work. Participants who experienced clinical symptoms suggestive of COVID-19 self-reported to the study investigators.

### Nasopharyngeal Sample Collection

Nasopharyngeal swabs (NPS) were collected at baseline and at follow-up visits following existing SPHMMC protocols for infection prevention to test for SARS-CoV-2 infection through real-time reverse transcription polymerase chain reaction (RT-PCR). NPS samples were collected using viral transport media, temporarily stored at 4°C, transferred to a −80°C freezer, and tested within 24 hours.

### Sample Processing

Viral RNA extraction was performed with MGISP-NE32 automated nucleic acid extraction technology [9] and amplified using the BGI real-time fluorescent RT-PCR kit [10]. A positive test result was defined by a cycle threshold value of 38 or less, with values that exceeded 38 reported as negative. Amplification reactions were carried out on the CFX96 Touch Real-Time PCR Detection System according to the manufacturer’s guidelines [11].

Incident SARS-CoV-2 infections are defined as new cases identified during the study period. These were detected through routine testing or symptom reporting and confirmed with positive results from RT-PCR assays. This includes both symptomatic and asymptomatic infections that occurred after initial participant enrollment and before the study concluded.

### Statistical Analyses

When designing this study, we targeted recruitment of 480 participants assuming 20% dropout during follow-up for a final sample size of 399. This sample size anticipated 30% of the cohort to be unvaccinated and an expected baseline frequency of SARS-CoV-2 infection of 24.4% in those unvaccinated [12] and 12.2% post-vaccination for a β = 0.20 and 2-sided α = 0.05.

The primary outcomes of interest were vaccination status and incident SARS-CoV-2 infection. Descriptive analyses were conducted for participant characteristics and outcomes. Categorical variables were summarized as frequencies and percentages, while the single continuous variable examined was reported as mean and range. Comparisons between groups were performed using the Fisher exact test for categorical variables, with odds ratios (ORs) and 95% confidence intervals (CIs) calculated to assess associations between participant characteristics and outcomes. For vaccination, ORs were presented as the increased odds of being unvaccinated compared to each reference group, while SARS-CoV-2 infection was assessed as a binary outcome (infected vs not infected).

All statistical analyses were performed using R (version 4.3.2; R Foundation for Statistical Computing, Vienna, Austria), and a 2-sided *P* value of <.05 was considered statistically significant.

### Study Approvals

The SPHMMC Institutional Review Board (IRB; PM23/383) and the Johns Hopkins School of Public Health IRB (IRB 17212; see 45 C.F.R. part 46; 21 C.F.R. part 56) approved the study.

## RESULTS

**Figure 1.**
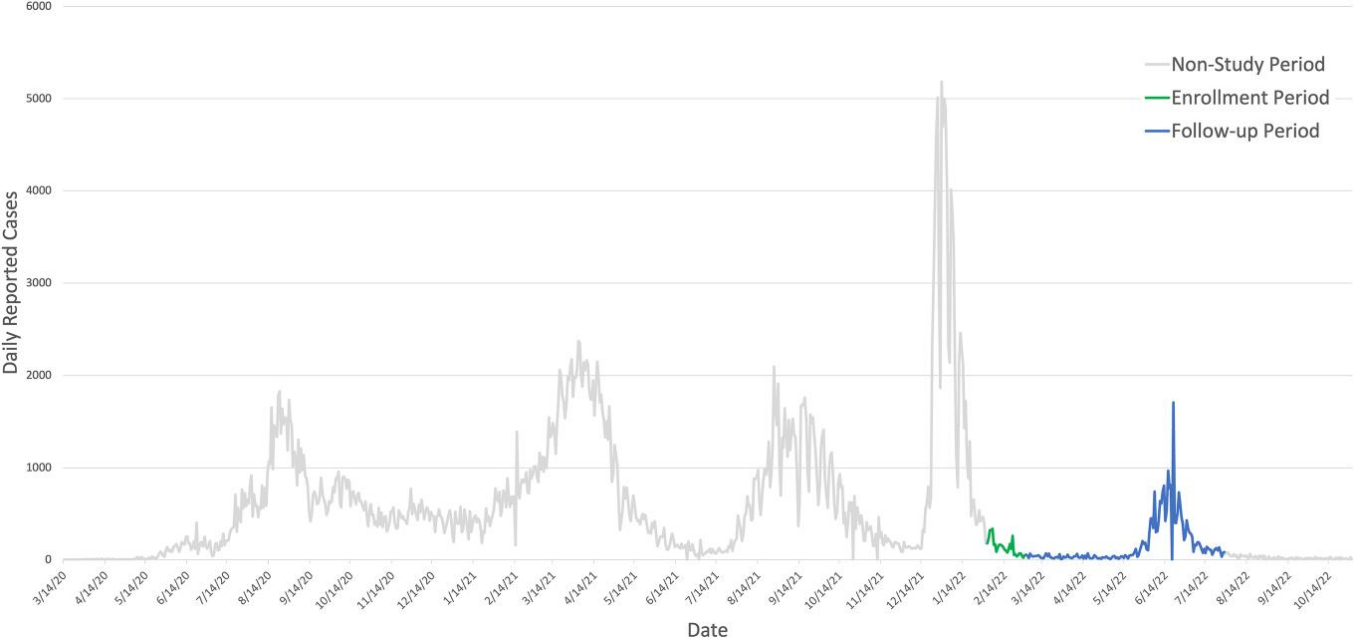
Daily reported coronavirus disease 2019 cases with study periods highlighted, Ethiopia (World Health Organization).

The demographic characteristics of the 469 enrolled participants are presented in Table 1. Baseline visits were conducted between late January 2022 through February 2022, immediately following a substantial wave of reported COVID-19 cases in Ethiopia (Figure 1). The time intervals and the proportion of the cohort with results available at each follow-up are provided here. It was approximately 3 weeks from enrollment to the first follow-up, with 90.6% (425 of 469) of the cohort participating. The interval from the first to the second follow-up remained at about 3 weeks, with the same participation rate of 90.6% (425 of 469). The interval between the second and third follow-ups extended to 4 weeks, with 58.6% (275 of 469) participating. From the third to the fourth follow-up, the interval increased to approximately 10 weeks, with 63.1% (296 of 469) of the cohort participating. Finally, from the fourth to the final follow-up, the interval was about 6 weeks, with 68.4% (321 of 469) of the cohort participating. In total, 34.9% (164 of 469) of participants completed all follow-up visits. Vaccination was widely available and promoted for HCWs throughout the study period.

**Table 1.** Association Between Select Participant Characteristics and Being Unvaccinated, Ethiopian Healthcare Worker Cohort, January 2022 to July 2022

Characteristic	Total N (%)	Unvaccinated n (%)^a^	Vaccinated n (%)	Odds Ratio (95% Confidence Interval)^b^	*P* Value
Total	469 (100)	202	267		
Sex					
Male	195 (41.6)	74 (37.9)	121 (62.1)	Reference	
Female	274 (58.4)	128 (46.7)	146 (53.3)	1.43 (.97, 2.12)	.07
Age category, y					
18–29	316 (67.4)	143 (45.3)	173 (54.7)	1.32 (.87–2.00)	.20
30–62	153 (32.6)	59 (38.6)	94 (61.4)	Reference	
Occupational group					
Physician	82 (17.5)	15 (18.3)	67 (81.7)	Reference	
Nurse	260 (55.4)	117 (45.0)	143 (55.0)	3.65 (2.03–6.95)	<.001
Patient transporter	29 (6.2)	19 (65.5)	10 (34.5)	8.49 (3.37–22.75)	<.001
Environmental Services	32 (6.8)	16 (50.0)	16 (50.0)	4.47 (1.85–11.08)	<.001
Other^c^	66 (14.1)	35 (53.0)	31 (47.0)	5.04 (2.45–10.82)	<.001
Working unit					
Dedicated COVID-19	30 (6.4)	8 (26.7)	22 (73.3)	Reference	
Emergency	74 (15.8)	39 (52.7)	35 (47.3)	3.06 (1.25–8.15)	.02
Gynecology/Obstetrics	56 (11.9)	24 (42.9)	32 (57.1)	2.06 (0.80–5.67)	.14
Internal medicine	48 (10.2)	18 (37.5)	30 (62.5)	1.65 (0.62–4.65)	.33
Intensive care	25 (5.3)	12 (48.0)	13 (52.0)	2.54 (0.84–8.11)	.11
Pediatrics	41(8.7)	13 (31.7)	28 (68.3)	1.28 (0.45–3.74)	.65
Surgical	24 (5.1)	12 (50.0)	12 (50.0)	2.75 (0.90–8.89)	.08
Addis Ababa Burn, Emergency, and Trauma Hospital (trauma center)	62 (13.2)	30 (48.4)	32 (51.6)	2.58 (1.03–6.98)	.05
Other^d^	109 (23.2)	46 (42.2)	63 (57.8)	2.01(0.85–5.17)	.13
Have you ever provided patient care to a suspected or confirmed COVID-19 case patient as part of your job?					
Yes: provided care	307 (65.5)	123 (40.1)	184 (59.9)	Reference	
No: never provided care	162 (34.5)	79 (48.8)	83 (51.2)	1.42 (.95–2.13)	.08
Other than in caring for a patient, have you ever been in close contact with a person you believe had COVID-19?					
Yes: have had contact	288 (61.4)	120 (41.7)	168 (58.3)	Reference	
No: no known contact	181 (38.6)	82 (45.3)	99 (54.7)	1.16 (.78–1.72)	.46
Do you feel that you and your colleagues are safe from contracting COVID-19 infection at work?					
Yes: feel safe	175 (37.3)	75 (42.9)	100 (57.1)	Reference	
No: do not feel safe	294 (62.7)	127 (43.2)	167 (56.8)	1.01 (.68–1.51)	1.00
Report of at least 1 prior positive COVID-19 test					
Yes: prior positive test	102 (21.7)	29 (28.4)	73 (71.6)	Reference	
No: no prior positive test	367 (78.3)	173 (47.1)	194 (52.9)	2.24 (1.37–3.75)	<.001

Abbreviation: COVID-19, coronavirus disease 2019.

^a^Unvaccinated includes participant reporting only a single dose of primary series.

^b^Odds ratios presented as increased odds of being unvaccinated compared to reference group.

^c^Other professions: pharmacy, laboratory technicians, data clerks.

^d^Other working unit: dermatology, psychiatry, human immunodeficiency virus treatment, otolaryngology, ophthalmology, kidney transplant, family medicine, anesthesiology, radiology. Working units represent a single health center.

### Vaccination

At baseline, 56.9% (267 of 469) of participants reported being fully vaccinated with a mean of 228 days (range, 59–352) from receipt of the second dose and study enrollment. For their primary series, most participants (265 of 267; 99%) received the Oxford-AstraZeneca vaccine, while 2 participants (1%) received the Janssen COVID-19 vaccine (Ad26.COV2.S). Only 1 initially unvaccinated participant became vaccinated during follow-up. Ten participants reported receiving a third dose or booster at baseline, while an additional 5 participants received a booster during follow-up. Among those who received booster doses, 12 received the Oxford-AstraZeneca COVID-19 vaccine and 3 received the Pfizer-BioNTech COVID-19 vaccine (BNT162b2).

Compared to other occupational groups, COVID-19 vaccination was more commonly reported in physicians (81.7% vs 51.7%; *P* < .001), and, while limited by the available sample, more staff assigned to dedicated COVID-19 patient care areas were vaccinated (73.3% vs 55.8%; *P* = .06). Vaccination was also more common among those who reported at least 1 prior positive COVID-19 test (71.6% vs 52.9%; *P* < .001).

Perceived workplace safety did not appear to drive vaccination decisions. The perception of increased COVID-19 risk in the workplace (*P* = 1.00) and providing care to suspected or confirmed COVID-19 patients (*P* = .08) were not statistically associated with vaccination status (Table 1). Notably, 62.9% (127 of 202) of unvaccinated individuals reported not feeling safe in the workplace yet still did not get vaccinated.

### Incident Infections

Over approximately 6 months of follow-up, 53 (53 of 469; 11.3%) incident SARS-CoV-2 infections occurred. No participants tested positive at enrollment, and no participant tested positive more than once. The following were not associated with testing SARS-CoV-2–positive (Table 2): COVID-19 vaccination (*P* = .66), sex (*P* = .56), age (*P* = .71), occupational group, working assignment, providing care (*P* = .08), and known exposure (*P* = .46) to known or individuals with suspected COVID-19. A perception of safety from SARS-CoV-2 infection in the workplace was associated with an increased likelihood of testing positive (OR = 2.05; 95% CI, 1.11–3.81; *P* = .02).

**Table 2.** Association Between Select Participant Characteristics and Severe Acute Respiratory Syndrome Coronavirus Positivity, Ethiopian Healthcare Worker Cohort, January 2022 to July 2022

Characteristic	Total N (%)	SARS-CoV-2–Positive n (%)	SARS-CoV-2–Negative n (%)	Odds Ratio (95% Confidence Interval)	*P* Value
Total	469 (100)	53 (11.3)	416 (88.7)		
Sex					
Male	195 (41.6)	24 (12.3)	171 (87.7)	1.19 (.67–2.19)	.56
Female	274 (58.4)	29 (10.6)	245 (89.4)	Reference	
Age category, y					
18–29	316 (67.4)	32 (10.1)	284 (89.9)	0.71 (.38–1.35)	.71
30–62	153 (32.6)	21 (13.7)	132 (86.3)	Reference	
Occupational group					
Physician	82 (17.5)	11 (13.4)	71 (86.6)	Reference	
Nurse	260 (55.4)	28 (10.8)	232 (89.2)	1.28 (.59–2.64)	.51
Patient transporter	29 (6.2)	4 (13.8)	25 (86.2)	0.97 (.30–3.74)	.96
Environmental services	32 (6.8)	2 (6.2)	30 (93.8)	2.32 (.58–15.61)	.29
Other^a^	66 (14.1)	8 (12.1)	58 (87.90	1.12 (.43–3.08)	.82
Working unit					
Dedicated COVID-19	30 (6.4)	4 (13.3)	26 (86.7)	Reference	
Emergency	74 (15.8)	10 (13.5)	64 (86.5)	0.98 (.25–3.24)	.98
Gynecology/Obstetrics	56 (11.9)	8 (14.3)	48 (85.7)	0.92 (.23–3.23)	.90
Internal medicine	48 (10.2)	5 (10.4)	43 (89.6)	1.32 (.30–5.44)	.70
Intensive Care	25 (5.3)	0 (0.0)	25 (100)	NA	NA
Pediatrics	41(8.7)	5 (12.2)	36 (87.8)	1.11 (.25–4.58)	.89
Surgical	24 (5.1)	1 (4.2)	23 (95.8)	NA	NA
Addis Ababa Burn, Emergency, and Trauma Hospital (trauma center)	62 (13.2)	6 (9.7)	56 (90.3)	1.44 (.34–5.47)	.6
Other^b^	109 (23.2)	14 (12.8)	95 (87.2)	1.04 (.28–3.2)	.94
Vaccination: report receipt of 2+ doses at least 14 days prior to enrollment					
Vaccinated: 2+ doses	267 (56.9)	32 (12.0)	235 (88.0)	1.17 (.63–2.22)	.66
Unvaccinated: none or single dose	202 (43.1)	21 (10.4)	181 (89.6)	Reference	
Have you ever provided patient care to a suspected or confirmed COVID-19 case patient as part of your job?					
Yes: provided care	307 (65.5)	36 (11.7)	271 (88.3)	1.13 (.60–2.23)	.76
No: never provided care	162 (34.5)	17 (10.5)	145 (89.5)	Reference	
Other than in caring for a patient, have you ever been in close contact with a person you believe had COVID-19?					
Yes: have had contact	288 (61.4)	28 (9.7)	260 (90.3)	0.67 (.36–1.25)	.18
No: no known contact	181 (38.6)	25 (13.8)	156 (86.2)	Reference	
Do you feel that you and your colleagues are safe from contracting COVID-19 infection at work?					
Yes: feel safe	175 (37.3)	28 (16.0)	147 (84.0)	2.05 (1.11–3.81)	.02
No: do not feel safe	294 (62.7)	25 (8.5)	269 (91.5)	Reference	
Report of prior positive COVID-19 test					
Yes: prior positive test	102 (21.7)	10 (9.8)	92 (90.2)	0.82 (.35–1.74)	.82
No: no prior positive test	367 (78.3)	43 (11.7)	324 (88.3)	Reference	

Abbreviations: COVID-19, coronavirus disease 2019; SARS-CoV-2, severe acute respiratory syndrome coronavirus 2.

^a^Other professions: pharmacy, laboratory technicians, data clerks.

^b^Other working unit: dermatology, psychiatry, human immunodeficiency virus treatment, otolaryngology, ophthalmology, kidney transplant, family medicine, anesthesiology, radiology. Working units represent a single health center.

## DISCUSSION

We examined the vaccination status and 6-month incident SARS-CoV-2 infections in a cohort of Ethiopian HCWs following multiple COVID-19 waves and widespread availability of safe and effective vaccination. Vaccine acceptance was modest, with only 56.9% uptake, varying by occupational group and assignment to dedicated COVID-19 patient care areas. Notably, vaccine uptake was not associated with HCW’s self-perception of infection risk or the recognition of providing care to patients with suspected or confirmed COVID-19. The vaccination rate observed in this cohort aligns with the suboptimal levels seen in the general Ethiopian population [6].

Despite better uptake among subgroups such as physicians (81.7%) and HCWs in COVID-19 units (73%), vaccine uptake remained suboptimal overall. The lack of association between vaccine uptake and both perceived and activity-based risks underscores the need for enhanced vaccine promotion strategies. Specifically, the finding that 62.9% of unvaccinated individuals reported not feeling safe in the workplace yet still did not get vaccinated underscores a critical disconnect. This might indicate a misconception about vaccine safety and efficacy, suggesting that concerns about workplace safety did not translate into higher vaccination rates.

Over 6 months of follow-up, 11.3% of the cohort experienced an incident SARS-CoV-2 infection. The symptoms reported were generally mild, and no HCWs required hospitalization for COVID-19. Incident infections did not correlate with any specific individual or occupational characteristics. However, a lower perceived risk of infection in the workplace was associated with a higher incidence of infection, suggesting a potential gap in risk perception and protective behaviors. Further investigation is needed to better understand factors that contribute to this association, such as adherence to infection prevention measures and workplace policies.

We faced challenges related to participant retention, as evidenced by a drop in participation rates from more than 90% to less than 60% by the third follow-up. Limited resources influenced these challenges, affecting the frequency and regularity of follow-ups, some of which extended up to 10 weeks. Such prolonged intervals might have led to fewer engaged participants, potentially resulting in missed infections and an underestimation of the 6-month incident RT-PCR positivity. To address these limitations, participants were encouraged to report any suspected episodes of illness for diagnostic testing, and periodic reminders were provided to enhance engagement and improve follow-up rates. The study also relied on self-reported data for vaccination status and suspected illness episodes, which could introduce recall bias or inaccuracies. To minimize self-report bias, we designed the questionnaire to avoid judgment regarding vaccination, ensured participants understood that vaccination was not mandatory, and emphasized that responses would remain confidential to encourage honest reporting.

Our findings highlight 2 critical educational opportunities for Ethiopian HCWs: understanding vaccine safety and effectiveness and accurately assessing infection risks. Addressing misconceptions about the vaccine is essential for improving vaccine uptake. Effective strategies to advocate for HCW vaccination may include engaging community leaders, such as religious leaders or workplace mentors, who can influence public opinion. These individuals can be identified through local networks and professional associations and engaged through collaborative workshops or training sessions focused on vaccine promotion. Additionally, tailored communication campaigns that address specific concerns and provide clear, evidence-based information about vaccine safety and efficacy can be delivered through accessible platforms, such as workplace seminars, healthcare facility communications, or social media channels. These approaches should remain adaptable to local needs and resources.

Improving vaccination coverage among HCWs requires a multifaceted approach. A 2020 systematic review and meta-analysis by Awoke and colleagues found that hepatitis B virus vaccination coverage among Ethiopian HCWs was low, with an overall uptake of only 20% (95% CI, 13.8–26.3) [13]. However, significantly higher uptake was observed among those with higher education levels, a personal history of exposure to blood and body fluids, and those who had received training in infection prevention [13]. Training on infection prevention and the risks associated with hepatitis B effectively increased vaccination coverage, underscoring the importance of comprehensive training programs and targeted strategies to address educational and experiential factors that influence vaccination uptake. While differences in vaccine uptake across occupational roles may stem from factors such as varying access to information, variations in perceived risk, or workplace-specific barriers, findings suggest the importance of adopting an approach that transcends occupational categories. Addressing overarching factors, such as education and engagement, through targeted strategies is critical to improving overall vaccine coverage.

Despite the end of the COVID-19 public health emergency declared by the WHO, prioritizing education in vaccination and risk assessment remains essential for preparedness against future public health emergencies. Infectious disease outbreaks challenge healthcare systems. Without appropriate management, healthcare facilities can become focal points for intensified disease transmission. A robust, well-informed, and well-vaccinated healthcare workforce is crucial for a successful public health response. Regularly monitoring vaccination rates and maintaining dedicated educational programs that emphasize the increased risk of exposure and disease transmission in healthcare settings are imperative.

Public health leaders and facility administrators should adopt a multifaceted approach to improve vaccination rates among HCWs. Regular training on infection prevention and control is crucial, emphasizing the role of vaccination in protecting HCWs, their families, and their communities [14]. Engaging trusted community and religious leaders to promote vaccination can help address hesitancy and misconceptions, fostering greater acceptance of health interventions [15]. Targeted communication campaigns that provide clear, evidence-based information can further increase vaccination rates by addressing barriers and misinformation [16].

Implementing these strategies can help build a robust, well-informed, and well-prepared healthcare workforce that is essential for a successful public health response and safeguarding the healthcare system against future public health emergencies.

## Notes

***Acknowledgments.*** The authors thank all St. Paul's Hospital Millennium Medical College healthcare employees who took part in this cohort study; their cooperation was greatly appreciated. The authors are also grateful to the microbiology laboratory staff for helping with real-time reverse transcription polymerase chain reaction testing.

***Financial support.*** This work was supported by the US Centers for Disease Control and Prevention (grant number: NU2HGH000007-135687).
